# Role of Acrostyle Cuticular Proteins in the Retention of an Aphid Salivary Effector

**DOI:** 10.3390/ijms232315337

**Published:** 2022-12-05

**Authors:** Maëlle Deshoux, Baptiste Monsion, Elodie Pichon, Jaime Jiménez, Aránzazu Moreno, Bastien Cayrol, Gaël Thébaud, Sam T. Mugford, Saskia A. Hogenhout, Stéphane Blanc, Alberto Fereres, Marilyne Uzest

**Affiliations:** 1 PHIM Plant Health Institute, Univ Montpellier, INRAE, CIRAD, Institut Agro, IRD, 34000 Montpellier, France; 2 Instituto de Ciencias Agrarias (ICA), Consejo Superior de Investigaciones Científicas (CSIC), Calle Serrano 115dpdo, 28806 Madrid, Spain; 3 John Innes Centre, Department of Crop Genetics, Norwich NR4 7UH, UK

**Keywords:** acrostyle, aphid effector, cuticular protein, feeding behavior, EPG, herbivore, insect–plant interactions, Mp10, stylet

## Abstract

To avoid the activation of plant defenses and ensure sustained feeding, aphids are assumed to use their mouthparts to deliver effectors into plant cells. A recent study has shown that effectors detected near feeding sites are differentially distributed in plant tissues. However, the precise process of effector delivery into specific plant compartments is unknown. The acrostyle, a cuticular organ located at the tip of maxillary stylets that transiently binds plant viruses via its stylin proteins, may participate in this specific delivery process. Here, we demonstrate that Mp10, a saliva effector released into the plant cytoplasm during aphid probing, binds to the acrostyles of *Acyrthosiphon pisum* and *Myzus persicae*. The effector probably interacts with Stylin-03 as a lowered Mp10-binding to the acrostyle was observed upon RNAi-mediated reduction in Stylin-03 production. In addition, Stylin-03 and Stylin-01 RNAi aphids exhibited changes in their feeding behavior as evidenced by electrical penetration graph experiments showing longer aphid probing behaviors associated with watery saliva release into the cytoplasm of plant cells. Taken together, these data demonstrate that the acrostyle also has effector binding capacity and supports its role in the delivery of aphid effectors into plant cells.

## 1. Introduction

Aphids are small insects of the order Hemiptera that can cause devastating crop losses [1]. These insects have piercing-sucking mouthparts composed of two pairs of stylets that navigate between and into plant cells and enable the establishment of a feeding site in the plant vascular bundle, primarily the phloem sieve elements (SE) [2]. This feeding behavior may cause direct damage to plant tissues and is also responsible for the transmission of a diversity of plant pathogens, including ~40% of all described plant viruses [3]. In addition, when feeding from the phloem, the large volume of sugary phloem sap ingested is mostly excreted as ‘honeydew’ that can become a substrate for the growth of sooty molds that prevent light absorption required for efficient photosynthesis of leaves. Despite this important burden imposed to crops, the molecular mechanisms that are essential for the establishment of a compatible aphid–plant interaction remain largely unknown.

More than 5500 aphid species have been described so far [4]. Of these, *Acyrthosiphon pisum* (pea aphid), which colonizes *Fabacea* [5] and *Myzus persicae* (green peach aphid), which colonizes over 400 plant species from 40 families [6], are the best-studied models. They transmit dozens of plant viruses and are extensively used to address diverse biological questions, including those related to plant–aphid defense/counter-defense processes.

Aphids secrete two types of saliva, gelling saliva and watery saliva. A small amount of gelling saliva, known as salivary flange, is produced on the surface of leaves at the site of stylet insertion into the leaf cuticle [7]. The gelling saliva is also secreted in the apoplast, where it forms a protective salivary sheath surrounding the stylet bundle and facilitating its gliding to deeper tissues [8]. Along the path to the vascular bundle, aphids use their stylets to probe the epidermis and mesophyll cells. Recently, Jimenez and colleagues [9] found that distinct brief (4–5 s) intracellular punctures may also occur in the phloem, in both companion and SE cells. During each probe, the stylets puncture through the cell membranes and reach the cell cytoplasm, where they first inject watery saliva, then acquire some cell content before they retract for navigation to the next cell. When stylets penetrate a SE cell, they remain inserted into the cell for longer, and this is associated with a long, watery saliva delivery phase followed by sustained phloem sap ingestion [10].

The gelling and watery saliva of aphids contain a diversity of proteins, including modulators of plant defense known as effectors [11]. Genome and transcriptome mining approaches and mass spectrometry analyses of saliva have contributed to the identification of numerous aphid effectors, of which only a few have been functionally characterized (described in [12,13]). Saliva effectors are often produced in salivary gland cells and released into the gland lumen that is connected to the salivary canal within the stylets. *A. pisum* C002 (ApC002) is one salivary gland protein that is abundantly present in plant tissues fed upon by aphids [14,15]. ApC002 is likely an effector given that the RNAi-mediated knockdown of *ApC002* causes reduced sustained phloem feeding and high aphid mortality [14,15]. Other salivary proteins such as Mp10 and PIntO1 in *M. persicae*, ACE1, Ap25 and Armet in *A. pisum* or MIF1 in both aphid species have been shown to modulate plant defenses and/or improve aphid performances [16,17,18,19,20,21,22]. Of these, *M. persicae* C002 (MpC002), PIntO1 (MpPIntO1) and Mp10 were detected around feeding sites by immunogold labeling of ultrathin plant tissue sections. In these sections, MpPIntO1 and MpC002 are found associated with the sheaths composed of gelling saliva in the apoplastic space, whereas Mp10 is detected in the cytoplasm of mesophyll cells that are in the immediate vicinity of aphid stylets, suggesting that Mp10 is delivered into these cells during a brief probe [23]. The intricate stylet behaviors and timely delivery of the watery saliva and Mp10 suggest the presence of a sophisticated effector delivery process.

Ultrastructure microscopy studies of aphid mouthparts revealed the existence of the acrostyle, a distinct anatomical cell-free cuticular structure located in the common food/salivary canal at the apex of the maxillary stylets [24], which typically penetrates the plant cell membrane and reaches the cell cytoplasm [25]. Five cuticular proteins, named Stylin-01 to Stylin-05, are located on the acrostyle surface. Studies of Stylin-01 and Stylin-02 demonstrated a role for Stylin-01 as a receptor of *Cauliflower mosaic virus* (CaMV) and the transmission of this virus [26,27]. CaMV is specifically retained on and released from the acrostyle within seconds [28,29]. It is likely that CaMV has adapted to use an existing structure and mechanism within aphid stylets for its own benefit. However, the function(s) of the acrostyle in the aphid feeding process, and more generally in plant–aphid interactions, has remained unclear.

Here, we show evidence that the acrostyle retains effector protein and provides experimental support for its role in effector delivery. Firstly, among the stylins, Mp10 specifically interacts with Stylin-03 in yeast two-hybrid experiments. Mp10 specifically binds to the acrostyle within the maxillary stylets as evidenced by epifluorescence microscopy. Furthermore, Mp10-binding to the acrostyle is reduced upon Stylin-03 RNAi, and aphids fed on Stylin-03 siRNA show altered feeding behavior, particularly in the early phases of plant–aphid interaction during the process of salivation into plant cells of both mesophyll and vascular tissues.

## 2. Results

### 2.1. Stylin-03 Interacts with the Effector Mp10 in Yeast

We previously identified five cuticular proteins (Stylin-01 through -05) in *A. pisum* anchored in the acrostyle, with one domain emerging at its surface, and thus accessible to salivary protein streaming down into the common food/salivary duct [27]. To investigate whether one or more of these five stylins bind aphid effectors, we conducted a yeast two-hybrid screen against characterized/validated *A. pisum* effectors available at the start of the project, including PintO1, C002, Armet, ACE1, Ap25, MIF1 and Mp10, hereafter referred to as Mp10_Apisum.

**Figure 1:**
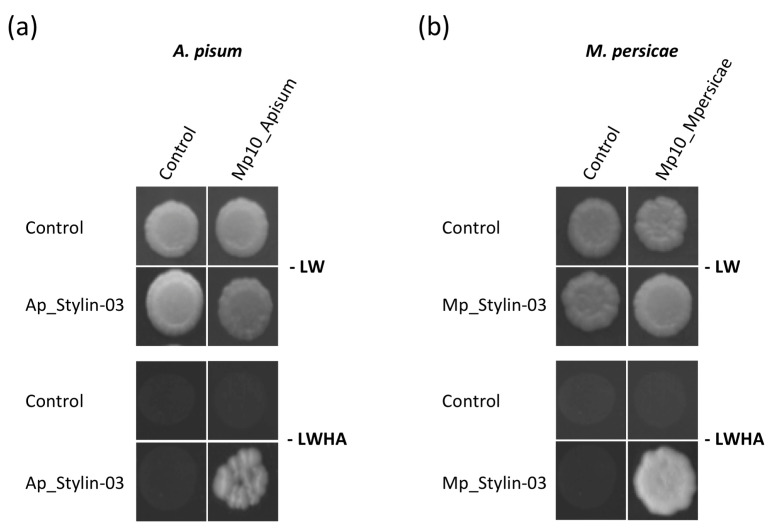
The cuticular protein Stylin-03 interacts specifically with the aphid effector Mp10 in yeast. NMY51 yeast strains carrying the bait vector pLexAN containing Ap_Stylin-03 (from *A. pisum*) (**a**), or Mp_Stylin-03 (from *M. persicae*) (**b**), were transformed with the prey vector pGADT7 containing a saliva effector of the same aphid species (Mp10_Apisum and Mp10_Mpersicae, respectively). Strains were spotted on synthetic defined (SD) non-selective medium (-LW, lacking leucine and tryptophan) and SD selective medium (-LWHA, lacking leucine, tryptophan, histidine, and adenine) and incubated at 28 °C for four days. The controls are the two plasmids pGADT7-GG-6His and pLexAN-GG-6His.

NMY51 yeasts co-transformed with bait pLexAN-stylin and prey pGAD-effector fusions could grow on SD [-LW] non-selective medium. On selective SD [-LWHA] medium, yeast growth was rescued when co-transforming Ap_Stylin-03 with Mp10_Apisum (Figure 1a). No interaction could be detected by this experimental system with the other *A. pisum* stylin/effector combinations used (Figure S1). Yeast growth was also rescued on a selective medium when co-transforming with Stylin-03 and Mp10 homologs of *M. persicae* (Mp_Stylin-03 and Mp10_Mpersicae, respectively; Figure 1b). We can thus conclude that Stylin-03 from both aphid species interacts with the corresponding Mp10 in the yeast two-hybrid system.

### 2.2. Mp10 Binds to the Acrostyle of A. Pisum and M. Persicae

**Figure 2:**
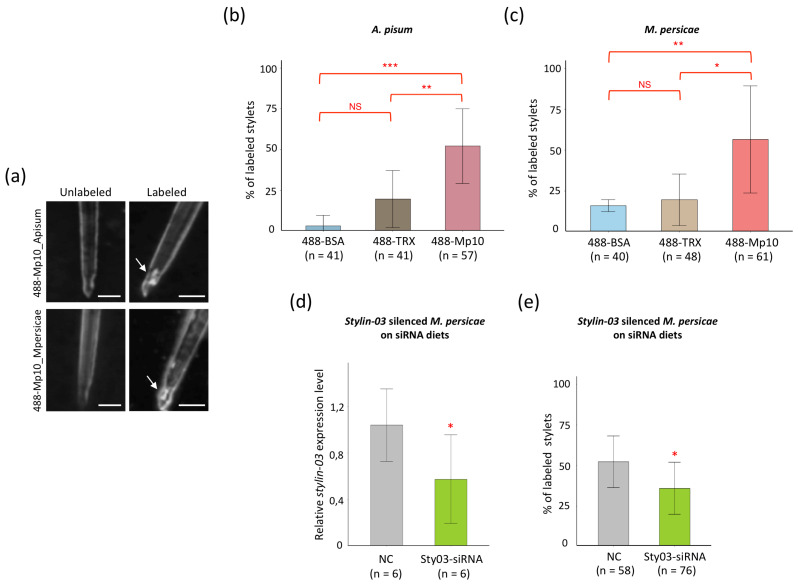
Mp10 directly binds to the acrostyle of dissected stylets. (**a**) Stylets of *A. pisum* and *M. persicae*, observed after incubation with 488-Mp10_Apisum and 488-Mp10_Mpersicae, respectively. Stylets were scored “labeled” when fluorescence was detected specifically at the apex of maxillary stylets, on the acrostyle (white arrow). Scale bars represent 5 µm. Proportion of *A. pisum* (**b**) or *M. persicae* (**c**) stylets showing fluorescence at the acrostyle area after incubation with Alexa Fluor 488 conjugates. Asterisks in (**b**) indicate a significant difference between 488-Mp10_Apisum and 488-BSA or 488-Mp10_Apisum and 488-TRX conditions (binomial GLM, Tukey tests, *p* < 1 × 10^−3^ and *p* = 4 × 10^−3^, respectively). 488-BSA and 488-TRX conditions do not differ significantly (Tukey tests, *p* = 0.089). Asterisks in (**c**) indicate significant difference between 488-Mp10_Mpersicae and 488-BSA or 488-Mp10_Mpersicae and 488-TRX conditions (Tukey tests, *p* = 4 × 10^−3^ and *p* = 0.013, respectively). 488-BSA and 488-TRX conditions do not differ significantly (Tukey tests, *p* = 0.76). (**d**) Transcript level of *stylin-03* in *M. persicae* treated with control (NC-siRNA) or specific Sty03-siRNA normalized against two reference genes. Data shown are the average of six pools of 10 aphids each. The asterisk indicates a significant difference compared to NC-siRNA control group (Welch’s *t*-test, *p* = 0.045). (**e**) Proportion of stylets of *M. persicae* treated with NC-siRNA or Sty03-siRNA showing fluorescence at the acrostyle area after incubation with 488-Mp10_Mpersicae. Asterisk indicates a significant difference between the two conditions (binomial GLM, *p* = 0.025). “n” in (**b**,**c**,**e**) indicates the total number of observed stylets for each treatment retrieved from three to five independent replicates. Bars represent standard deviations. Asterisks indicate statistically significant differences as follows: ***, *p* < 0.001; **, *p* < 0.01; *, *p* < 0.05. NS—not significant.

Because Stylin-03 was detected at the acrostyle surface [27], Mp10 may locate at the acrostyle as well. To test this, we produced mature (without signal peptides) untagged Mp10_Apisum and Mp10_Mpersicae proteins in *Escherichia coli* (Figure S2) and conjugated the purified Mp10 proteins to Alexa Fluor 488 to generate 488-Mp10_Apisum and 488-Mp10_Mpersicae. The Thioredoxin (TRX), derived from the plasmid used to produce Mp10 proteins, and the bovine serum albumin (BSA, Promega Corporation, Madison, Wisconsin, USA), a non-aphid protein, were also conjugated to Alexa Fluor 488 (488-TRX and 488-BSA, respectively) to assess the non-specific binding threshold of any Alexa Fluor 488 conjugate in the experimental conditions used (see Section 4.4). About 10 µg of the different conjugates were incubated with dissected aphid stylets using a protocol that previously showed binding of CaMV to aphid acrostyles [29]. Because fluorescence on dissected stylets is hardly quantifiable [26], stylets were scored “labeled” when fluorescence was detected specifically on the acrostyle, regardless of the intensity of the signal. To generate statistically meaningful data, for each series of experiments, all stylets were observed using identical microscope settings. Of the 57 *A. pisum* stylets incubated with 488-Mp10_Apisum, 29 (51%) showed fluorescence specifically at the acrostyle region (Figure 2a,b), as opposed to 7 out of 41 (17%) and 1 out of 41 (2%) of the stylets incubated with 488-TRX and 488-BSA, respectively (Figure 2b). Similarly, 34 out of 61 (56%) of the *M. persicae* stylets incubated with 488-Mp10_Mpersicae showed fluorescence specifically at the acrostyle region (Figure 2a,c), as opposed to 10 out of 48 (21%) and 6 out of 40 (15%) of the stylets incubated with 488-TRX and 488-BSA, respectively (Figure 2c). These data show that 488-Mp10 labeled significantly more stylets at acrostyle regions than control 488-BSA and 488-TRX.

To examine whether Stylin-03 at the surface of the acrostyle mediates Mp10 binding at the acrostyle, 488-Mp10_Mpersicae binding to dissected stylets was compared in wild-type versus Stylin-03-RNAi *M. persicae*. Stylin-03 expression was reduced by about 50% on average (Figure 2d). 488-Mp10_Mpersicae labeled 31 out of 58 (53%) of the acrostyles of wild-type aphids (Figure 2e), consistent with previous results (Figure 2c), indicating that feeding on non-matching siRNA has no noticeable effect on acrostyle binding capacity. In contrast, 488-Mp10_Mpersicae only labeled 26 out of 76 (34%) of those of Stylin-03-RNAi *M. persicae* (Figure 2e). These data indicate that Mp10 is less likely to bind acrostyles if the level of Stylin-03 is reduced in aphids, consistent with the hypothesis that Stylin-03 recruits Mp10 to the acrostyle surface.

### 2.3. Impact of siRNA Targeting Stylin-03 or Stylin-01 Gene Expression on M. Persicae Feeding Behavior

**Figure 3:**
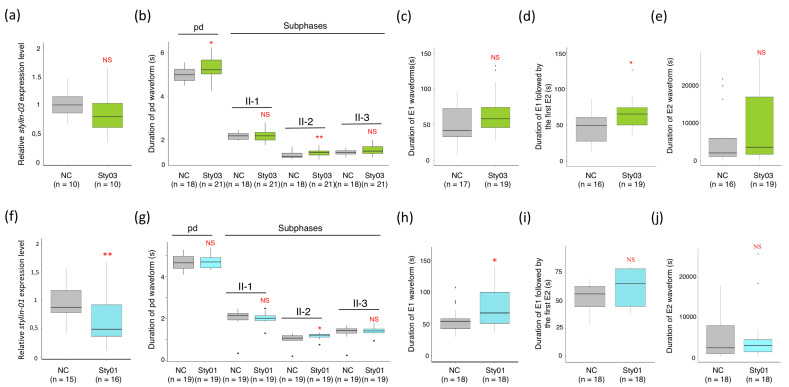
Boxplots showing the impact of RNAi-mediated silencing of *stylin-03* and *stylin-01* on *M. persicae* feeding behavior on *Arabidopsis thaliana* plants. (**a**,**e**) Results associated with *stylin-03* gene knockdown (Sty03) compared to the control groups (NC). (**f**,**j**) Results associated with *stylin-01* gene knockdown (Sty01) compared to the control groups (NC). The transcript level of *stylin-03* (**a**) or *stylin-01* (**f**) were normalized against two reference genes (*actin* and *EF1α*). The relative expression of *stylin-03* was not significantly affected (*n* = 10 pools of 10 aphids each, Welch’s *t*-test, *p* = 0.49) (**a**), while the relative expression of *stylin-01* was significantly reduced (*n* = 15 pools of 10 aphids for control groups (NC) and *n* = 16 pools of 10 aphids each for Stylin-01-silenced aphids each; Welch’s *t*-test, *p* = 0.00445) (**f**). (**b**,**g**) Mean duration of intracellular punctures (potential drops, pds) and pd sub-phases (II-1, II-2, II-3). The mean duration of pd increased in Stylin-03-silenced aphids, due to a significant increase in sub-phase II-2 (**b**). The duration of sub-phase II-2 was also significantly longer in Sty-01-siRNA-treated aphids, though the total duration of the pd was not different from control groups (**g**). (**c**,**h**) Mean duration of E1 waveform, associated with salivation into phloem sieve elements. The mean duration of E1 increased for treated aphids when compared with control groups. However, this difference was significant only for Stylin-01 silencing experiments (**h**), not for Stylin-03 silencing experiments (**c**). (**d**,**i**) Mean duration of E1 followed by the first E2. This variable increased significantly for Stylin-03-silenced aphids when compared to the control groups (**d**). No significant difference was observed between the two groups in Stylin-01 silencing experiments (**i**). (**e**,**j**) Mean duration of E2 waveform associated with passive ingestion of phloem sap. No significant difference was observed between the two groups. The number (*n*) of aphids recorded for each EPG variable is indicated below the x-axis. All values are reported in Supplemental Tables S1 and S2. Asterisks indicate statistically significant differences as follows: **, *p* < 0.01; *, *p* < 0.05. NS—not significant.

The observations described above and in an earlier report demonstrate that at least two stylins have the capacity to interact with proteins streaming up and/or down at the surface of the acrostyle surface: Stylin-03 binds to the saliva effector Mp1, and Stylin-01 likely binds to the viral protein P2 of CaMV [26]. To investigate a possible role of these two stylins in the feeding process (beyond CaMV P2, Stylin-01 could also bind to unidentified partners), we knocked down their expression in *M. persicae* one-to-three-day-old nymphs. The N4 nymphs emerging from these treated individuals were transferred to new plants for electrical penetration graph (EPG) monitoring of their feeding behavior [10,30], a process previously optimized in our laboratory [26]. Several sequential and non-sequential variables, including probing, salivation into SE and passive uptake of phloem sap, were analyzed (Figure 3 and Supplemental Tables S1 and S2).

While in this series of experiments RNAi treatments had no detectable impact on the *stylin-03* transcripts (Figure 3a; see Discussion), *stylin-01* transcripts were significantly reduced by 38% (Figure 3f). Noticeably, despite contrasted effects on stylin transcripts, some EPG variables were impacted in the two cohorts fed on Stylin-siRNAs (Figure 3). The duration of intracellular punctures (recorded as potential drops, i.e., ‘pd waveform’) was significantly longer for Sty03-siRNA-treated aphids, due to an increase in the mean duration of sub-phase II-2 (1.41 ± 0.04 s vs. 1.28 ± 0.04 s), whereas sub-phases II-1 and II-3 remained unchanged (Figure 3b). The duration of the entire intracellular puncture was not impacted for the Sty01-siRNA-treated aphids. However, sub-phase II-2 was also significantly longer for the silenced aphids when compared with the control groups (1.14 ± 0.03 s vs. 1.01 ± 0.07 s) (Figure 3g). The function of sub-phase II-2 is unknown [31], but this sub-phase has been associated with CaMV release into plant cells, and, thus, presumably with intracellular salivation [32]. Interestingly, variables related to salivation into the phloem SE were also significantly impacted. The mean duration of E1 (salivation into SE) followed by the first E2 (phloem sap ingestion) increased by 39.5% for Sty03-siRNA-treated aphids (79.02 ± 15.34 s vs. 56.65 ± 12.56 s for controls) (Figure 3d), and the mean duration of E1 waveform was more than 30% longer in Stylin-01-silenced aphids (74.19 ± 7.39 s vs. 56.11 ± 4.65 s for controls) (Figure 3h). Finally, the time spent in phloem-sap ingestion (E2 waveform) was not significantly affected for both cohorts (Figure 3e and Figure 3j, respectively).

### 2.4. Impact of siRNA Targeting Stylin-03 or Stylin-01 Gene Expression on M. Persicae Survival Rate and Fecundity

**Figure 4:**
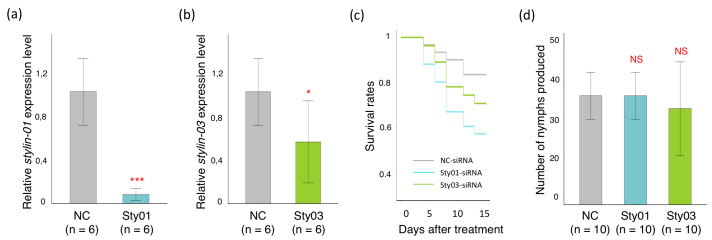
Silencing *stylin-01* or *stylin-03* in *M. persicae* has a contrasting impact on aphid fecundity and longevity. The transcript level of *stylin-01* (**a**) or *stylin-03* (**b**) normalized against two reference genes (*actin* and *EF1α*). (**a**) The relative expression level of *stylin-01* is significantly reduced by 95% in treated *Myzus persicae* (Sty01) compared with the control aphids (NC) (Welch’s *t*-test, *p* = 5.2 × 10^−4^). (**b**) Identical to Figure 2d, with fecundity and longevity monitored on aphids from the same treated cohorts as the ones used for stylet dissection. The relative expression level of *stylin-03* (Sty03) is significantly reduced by 46% compared with the control aphids (Welch’s *t*-test, *p* = 0.045). (**c**) Survival curves of treated aphids. The different lines represent the survival curves for the three groups of aphids. The results are reported as means (initial *n* = 30, 25 and 27 aphids for NC, Sty01 and Sty03 groups, respectively). (**d**) A number of nymphs are produced for each condition. Data shown are means (± standard deviation) of total number of nymphs produced over 15 days per apterous adult (*n* = 10). No significant difference was observed between the three groups (ANOVA, 0.45 < *p* < 1). Asterisks in panels A and B indicate statistically significant differences as follows: ***, *p* < 0.001; *, *p* < 0.05. NS—not significant.

To investigate whether RNAi treatments impacted aphid life traits, we assessed the fecundity and survival rate of silenced and control aphid groups. In this experiment, the accumulation of *stylin-01* and *stylin-03* transcripts significantly decreased by 95% for aphids fed with Sty01-siRNA and by 46% for those fed with Sty03-siRNA (Figure 4a,b).

Our results revealed contrasted impacts of RNAi treatments on the two aphid life traits. Both Stylin-silenced groups showed higher mortality than the NC control group during the 15-day follow-up period, with the highest mortality observed for Stylin-01-silenced aphids (Figure 4c). In contrast, the treatments did not impact the fecundity of viable mothers, which remained similar in all groups. Thus, the total number of nymphs produced by 10 apterous aphids over the 15 days of recording were similar in the three groups, with 36 ± 1.9 nymphs produced for NC control and Stylin-01-silenced aphids, and 32.7 ± 3.8 nymphs produced for Stylin-03-silenced aphids (Figure 4d).

## 3. Discussion

The acrostyle, discovered in 2010, is a well-defined anatomical structure with unique surface properties differing from the rest of the cuticle of aphid stylets [24,27]. This organ is restricted to a region of the maxillary stylets, downstream the food and salivary canals, and is in contact with diverse compounds from the sap streaming up and from the saliva streaming down. It was first characterized because of its ability to interact with the viral protein P2 ensuring CaMV retention during its transport from one plant to another [29]. CaMV is transmitted in a noncirculative manner. Its transient binding to the acrostyle occurs in brief (a few seconds) intracellular punctures (recorded as potential drops or pds) in infected cells [33]. The release of CaMV from aphid stylets is also a fast process—the virus is inoculated into plant cells during the first brief intracellular puncture [32]. Beyond virus transmission, the role of the acrostyle for aphid physiology was totally unexplored. Recent results on its surface composition, however, provided new opportunities to investigate its function, through the search for compounds that may bind stylins, just as viral proteins do [26,27].

In this study, we showed that the effector Mp10 is retained at the surface of the acrostyle of dissected stylets through direct interaction with a protein of this cuticular organ, in the two aphid species investigated. Our yeast two-hybrid assay pointed to Stylin-03 as the cuticular protein mediating this interaction. Unfortunately, a direct demonstration is not experimentally accessible; therefore, indirect evidence is necessary and most valuable. An approach commonly used to investigate gene functions in organisms is the reduction of their expression through RNA interference. We here applied this technology to reduce the amount of Stylin-03 in the acrostyle. However, silencing in aphids is notoriously poorly efficient, highly variable, transient and incomplete [34,35,36]. As such, realistically, we were not expecting to obtain a Stylin-03-free acrostyle and a complete loss of its Mp10-binding capacity upon silencing treatment, but rather a variable reduction in the number of corresponding binding sites and consequently rarer detectable labeling of Mp10 at the tip of dissected stylets, which we observed. Another important issue is that the efficiency of silencing stylin genes in the targeted tissue cannot be easily evaluated. A reduction of the protein amount in the acrostyle was not directly quantifiable due to the size of this structure and the difficulty of extracting proteins from the stylets [27]. A quantification of mRNA levels in stylet-secreting glands (retort organs) is not achievable either. Here, we evaluated stylin transcript levels in whole insects as a proxy. However, stylins are not stylet-specific proteins. They are expressed in different body parts [27]. Moreover, RNAi efficiency can vary considerably between aphid tissues [27,37]. We are aware that these snapshot quantifications may not always faithfully reflect what happens in stylet-secreting glands during the unknown timeframe of acrostyle synthesis, especially because we have shown in a previous study that stylin transcript levels are highly variable during aphid development [38]. All these unavoidable drawbacks call for a cautious interpretation of mRNA quantifications. Nonetheless, the ingestion of siRNA specifically targeting *stylin-03* mRNA resulted in an acrostyle binding-deficient phenotype, thereby strongly suggesting Stylin-03 as the acrostyle Mp10-binding protein, although, at this stage, the involvement of another stylin cannot be completely ruled out.

Why would the acrostyle retain Mp10? Mp10 belongs to the chemosensory protein family, also known as OS-D-like proteins [16]. Mp10 is known to suppress the reactive oxygen species (ROS) burst triggered specifically by the bacterial MAMP flg22 [16], suggesting that this effector could be a suppressor of PTI (Plant-Triggered Immunity) in plants. Mp10 is a conserved effector gene, with low evolutionary rate, and could be involved in fundamental functions in the modulation of host plant immunity [12,39]. Mp10 is excreted in defined target tissues [23] suggesting it is released at a specific step of the aphid feeding process. If space and time regulations are mandatory for Mp10 to be effective in plant tissue, then binding transiently to the acrostyle may allow such accurate time-space delivery and ensure optimized alteration of Mp10-targeted plant defenses. In addition, not excluding the hypothesis suggested above, we speculate that binding to the acrostyle could help accumulate Mp10 prior to its release in the target tissue, thereby reaching the concentration threshold required for effective action in plant cells. According to the above hypotheses, Stylin-03-silenced aphid behavior may be altered in the early phases of the feeding process when the insect assesses whether or not the plant is suitable for sustainable phloem sap uptake.

We have recorded the behavior of Stylin-03-silenced aphids with a particular focus on the key phases of the feeding process during which Mp10 is normally delivered. Although silencing efficiency was apparently not affected in these experiments, aphids fed on Stylin-03 siRNA showed longer activities during intracellular punctures, subphase II-2. The effect was even stronger when the insects reached the phloem SE, where the salivation phase E1 corresponding to the delivery of watery saliva was increased by 39.5% before starting the first phloem sap ingestion phase (E2). Extended E1 salivation phases have already been reported in phloem-based resistance to aphids [10,40,41]. This increase in the E1 salivation phase generally indicates that aphids encounter difficulties in the initiation of phloem sap ingestion. Aphids tend to extend their salivation into SE to counteract host plant defense mechanisms, including SE occlusion or after leaf-tip burning [42]. Interestingly, we did not observe any change during the subsequent E2 phase of passive ingestion of phloem sap, suggesting that the behavioral changes recorded in our experiments do not reflect a general alteration of the insect’s physiological condition, but a very specific effect on early plant–aphid interactions. Sty03-siRNA-treated aphids resume a normal and sustainable phloem sap ingestion suggesting that longer salivation fully compensates for the acrostyle defect in Mp10-binding capacity. Of important note is the fact that the acrostyle surface displays several stylins from the same subfamily (CPR_RR-1 proteins, Cuticular Protein with a Rebers and Riddiford consensus sequence), some of which have a high level of identity with possible functional redundancy [26,27]. Thus, other stylins may partly compensate for a reduced amount of Stylin-03 at the surface of the acrostyle. Consistently, we also observed changes in aphid salivation phases after silencing Stylin-01, another acrostyle surface protein. This observation indicates that regardless of the stylin targeted, feeding aphids on specific siRNAs results in altered phases of the aphid feeding behavior related to salivation. In addition, the stylin-siRNA treatments also had a negative effect on aphid survival rate. Although it would be tempting to conclude that these altered phenotypes could be a consequence of the disruption of acrostyle function. However, for the reasons mentioned above, we cannot rule out at this stage indirect effects and further investigations would be required.

**Figure 5:**
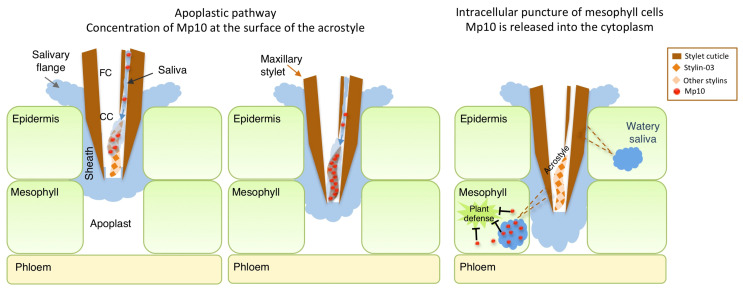
Model for the regulation of Mp10 delivery through acrostyle binding. One maxillary stylet tip inside plant tissue is represented in brown. The food canal (FC), common canal (CC) and acrostyle are indicated. During the apoplastic pathway, Mp10 (red dots) contained in gelling saliva (light blue) would bind to Stylin-03 (orange diamonds) at the surface of the acrostyle. While puncturing cells along the stylet track (pictured as brown dotted lines), watery saliva (dark blue) is secreted and flushed out. All of the Mp10 accumulated on the acrostyle would then be released in mesophyll cells where effector molecules can modulate plant responses.

Based on our results, we propose a model in which the acrostyle would act as a regulator of Mp10 delivery into the cytoplasm of mesophyll cells, where the effector has been detected [23], and hence would participate actively to plant–aphid compatible interactions (Figure 5). Mp10 would be secreted in the first phases of the aphid feeding process together with the gelling saliva that forms the salivary flange or the salivary sheath. While the stylets navigate into the apoplast, some of the egested Mp10 molecules would bind at least to Stylin-03 at the surface of the acrostyle. When the stylets penetrate plant cells for probing cell content, all bound Mp10 would be released from the acrostyle and flushed out into the cytoplasm along with watery saliva. Disruption of effector-stylin interaction may be favored by local changes in saliva composition or a shift in the pH [8] that could induce conformational changes of Mp10. Chemosensory proteins have been shown to exhibit drastic conformational changes when bound to their ligand [43].

Interestingly, beyond aphid acrostyle at the tip of maxillary stylets, a cuticular structure known as V-shaped ridges located at the tip of the stylet fascicle of Aedes mosquitoes was recently shown to exhibit salivary protein-binding capacity [44]. In their study, the authors demonstrated that LIPS-2, the labrum-interacting protein of the saliva 2 of Aedes mosquitoes, interacts with Cp19, a cuticular protein from the CPR_RR-2 subfamily emerging on the external cuticle of the apical part of the labrum. Fluorescence signals from a GFP-fused LIPS-2 variant incubated on dissected stylets from mosquitoes were strictly restricted to the labral ridges. Remarkably, the exposition to recombinant LIPS-2 protein induced morphological changes in the ridges that appeared taller than those of non-salivating mosquitoes, and stimulated probing events. Consistently, LIPS-2 knockdown females showed increased intradermal probing prior to blood engorgement. LIPS-2 is a female-specific salivary factor shown to have immunogenic properties in humans [45]. Arnoldi and colleagues characterized a new role for this protein in establishing a feedback signaling pathway in aedines, and further showed that interaction with Cp19 is the initial trigger of this signaling pathway [44]. Echoing our present study, this article highlights that the cuticular proteins at the tip of piercing-sucking insects can act as a natural platform for key molecular interactions, in this case, with a mosquito salivary protein involved in the initial phases of biting behavior.

Altogether, our results support a role for the acrostyle in effector delivery, Mp10 being the first effector shown to bind to the acrostyle. It will be interesting to further investigate if other effectors are retained in the same way or interact with other stylins. Our previous studies already demonstrated that the acrostyle harbors virus receptors at the surface of the stylet cuticle [24,26,29]. Here, we reveal another facet of its binding capacity and provide evidence for its functional role for the aphid, i.e., the delivery of an effector modulating plant defenses.

## 4. Materials and Methods

### 4.1. Aphid Colonies

All aphid species were reared in an environmental growth chamber at a temperature of 23/18 °C and a photoperiod of 14/10 h (day/night). *Myzus persicae* (Sulzer) and *Acyrthosiphon pisum* (LL01) colonies were maintained on *Solanum melongena* cv. Barbentane and on *Vicia faba* cv. Aguadulce, respectively.

### 4.2. Yeast Two-Hybrid Constructs and Assays

Cuticular proteins of the acrostyle used in this study were: Stylin-01 (ACYPI009006), Stylin-02 (ACYPI003649), Stylin-03 (ACYPI001610), Stylin-04 (ACYPI002877) and Stylin-05 (ACYPI007911) from *A. pisum* and their orthologs in *M. persicae*. Genes encoding mature proteins without signal peptides were obtained from cDNA amplification.

Open reading frames (ORFs) of *A. pisum* effectors lacking the signal peptide sequences used in this study were amplified from various sources: Armet (ACYPI008001) and ACE1 (ACYPI000733) from plasmids kindly provided by Dr. Feng Cui [20,21]; Ap25 (ACYPI009919) from a plasmid kindly provided by Dr. Akiko Sugio [22]. Sequences of C002 (ACYPI008617) [14], MIF1 (ACYPI002465) [19], PintO1 (ACYPI6346) [17] from *A. pisum*, and Mp10 from *M. persicae* were synthesized and cloned in plasmid-based transfer vectors by Proteogenix (Schiltigheim, France). In addition, Mp10 *A. pisum* homolog was amplified from *A. pisum* cDNA. For clarity, in this article, these two last effectors are respectively named Mp10_Mpersicae and Mp10_Apisum.

All sequences were PCR-amplified using Phusion High-Fidelity DNA Polymerase (Thermo Fisher Scientific, Rockford IL, USA) and specific primers containing SapI restriction site at their 5′ end (listed in Supplementary Table S3). They were further cloned in pGADT7 (Clontech, Palo Alto, CA, USA) and pLexAN (Dualsystems Biotech, Zurich, Switzerland) modified plasmids (modifications described in Supplementary Information). Effector sequences were introduced downstream of the GAL4 activation domain (AD) in the pGADT7-GG plasmid, while stylin sequences were introduced downstream of the LexA binding domain in the pLexAN-GG plasmid using Sap1 restriction sites. The two plasmids, pGADT7-GG-6His (six histidine residues fused to AD) and pLexAN-GG-6His, (six histidine residues fused to the DNA-binding domain) were used as negative controls. All constructs were confirmed by sequencing.

pGADT7-GG and pLexAN-GG derived-constructs were co-transformed in the NMY51 yeast strain [46]. Interactions were identified following the procedures described in the DUALhunter kit user manual (Dualsystems Biotech). Co-transformants were first selected onto a synthetic-defined (SD) medium lacking leucine and tryptophan [-LW]. Colonies were then screened on a selective SD medium lacking leucine, tryptophan, histidine, and adenine [-LWHA] to identify interactions. Petri dishes were incubated at 28 °C for four days. Four independent experiments were performed.

### 4.3. Constructs, Production and Labeling of Mp10 for Detection on Insect Stylets

Sequences encoding Mp10_Mpersicae or Mp10_Apisum mature proteins, amplified by PCR as described above, were cloned in plasmid pETtrx1b (EMBL, Heidelberg, Germany) in fusion with a histidine tag and the gene encoding Thioredoxin (His-TRX-Mp10), using XhoI and NcoI restriction sites. All constructions were verified by sequencing. His-TRX-Mp10 fusions were produced in *E. coli* BL21 strain by induction of bacterial cultures at OD600 = 0.6 with 0.5 mM IPTG (isopropyl β-D-1-thiogalactopyranoside) for 3 h at 37 °C. Cell pellets were resuspended in 15 mL of phosphate-buffered saline buffer [4.3 mM Na2HPO4, 1.4 mM KH2PO4, 137 mM NaCl, 2.7 mM KCl, pH 7.3] supplemented with 1 mM Dithiothreitol (PBS-DTT buffer) and 10 mM imidazole, sonicated, and incubated for 1 h at 4 °C. Cell debris were removed by centrifugation for 10 min at 12,000 g. The soluble fraction was stirred for 1 h with 2 mL of Ni-nitrilotriacetic resin (Qiagen, Hilden, Germany) previously equilibrated with PBS-DTT buffer, transferred to a 5 mL column, and rinsed with 10 volumes of PBS-DTT buffer. Mp10 was finally eluted using 2 mL of PBS-DTT buffer supplemented with 500mM imidazole, dialyzed overnight in PBS-DTT buffer, and cleaved from the histidine tag and thioredoxin using AcTEV protease (Thermo Fisher Scientific, Rockford IL, USA) according to the manufacturer’s instructions. Purified Mp10 was adjusted to a final concentration of 1 mg/mL, and further conjugated to Alexa Fluor 488 dye using Alexa Fluor 488 Microscale Protein Labeling Kit (Molecular Probes, Eugene, OR, USA) according to the manufacturer’s instructions. In addition, two proteins, used as negative controls in our experiments were conjugated as described above, the BSA (Promega Corporation, Madison, WI) and the His-TRX, derived from the same plasmid used to produce Mp10.

### 4.4. In Vitro Interactions between Mp10 and Dissected Aphid Stylets

Stylets from adult aphids were dissected, individualized and placed on siliconized cover slides as previously described [29]. The stylets from *M. persicae* or *A. pisum* were incubated with 10 µg of Alexa Fluor 488-conjugated Mp10_Mpersicae (488-Mp10_Mpersicae) or with Mp10_Apisum (488-Mp10_Apisum), respectively, in 290 µL of MES buffer (50 mM MES [2-(N-morpholino) ethanesulfonic acid], pH 5.8, supplemented or not with 1 mM DTT) for an incubation period of 2–4 h at room temperature in the dark. To maximize the chance of observing even labile interactions, we specifically designed a one-step incubation protocol and avoided extensive rinses. In parallel, Alexa Fluor 488-conjugated His-TRX (488-TRX) or Alexa Fluor 488-conjugated BSA (488-BSA) were incubated onto *A. pisum* and *M. persicae* stylets as controls. In all experiments, stylets were observed with an Olympus BX60 microscope equipped for epifluorescence using identical microscope settings, and scored “labeled” when fluorescence was detected specifically on the acrostyle, regardless of the intensity of the signal. Between three and five independent replicates were performed with a total of 41 to 61 stylets observed for each condition (488-Mp10_Apisum or 488-Mp10_Mpersicae, 488-TRX, 488-BSA) and each aphid species (*A. pisum* and *M. persicae*). In addition, stylets from the control and Sty03-RNAi-treated aphids (*M. persicae*) were incubated with 488-Mp10_Mpersicae. In these experiments, N3-synchronized nymphs were fed for 48 h on siRNA artificial diets (see below for RNAi experiments). In these conditions, the aphid nymphs became adults with their definitive stylets at the end of the treatment. Four independent experiments were performed, and a total of 58 to 76 stylets were observed.

### 4.5. RNA Interference

To silence stylin-03 and stylin-01 gene expressions, short interfering RNA (siRNA) specifically targeting one or the other mRNA (Sty03-siRNA and Sty01-siRNA, respectively, Kanaka Eurogentec S.A.; see sequences in Supplemental Table S3) or negative control siRNA (NC-siRNA, SR-CL000-005, Eurogentec) were delivered through a sachet to cohorts of *M. persicae* aphids at a final concentration of 1 µM in the diet, as previously described [26]. As the maxillary stylets are cell-free tissues, RNAi treatments were applied to early nymph stages to target the stylet-secreting glands during stylet synthesis. The nymphs were fed for 48 h on siRNA artificial diets. Phenotypes were evaluated at least after one molting event post-siRNA delivery at N4 or adult stage.

### 4.6. Quantitative PCR 

Although they may not reflect silencing in retort organs, (where stylets are synthesized), the stylin transcript levels were quantified in whole insects as a proxy for silencing efficiency. Aphids were collected after 48 h on siRNA artificial diets. Total RNA was extracted from pools of ten aphids using the RNAeasy mini kit (Qiagen, Hilden, Germany). Total RNA (300 ng) was then treated with RQ1 RNase-Free DNase I (Promega Corporation, Madison, Wisconsin, USA) and reverse-transcribed using the Moloney murine leukemia virus (MMLV) reverse transcriptase (Promega Corporation, Madison, Wisconsin, USA) and oligo(dT) as a primer. Quantitative PCR was performed in duplicates on a LightCycler 480 instrument using a LightCycler 480 SYBR green master mix (Roche, Penzberg, Germany) according to the manufacturer’s recommendations. Data shown in Figure 2d and Figure 4b resulted from the analyses of 6 pools of 10 aphids each, and the ones shown in Figure 3a,f resulted from the analyses of 10 pools of 10 aphids each. The gene-specific primers used are listed in Supplemental Table S3. Amplification efficiencies were analyzed with the LinRegPCR software (v. 2014.5) [47]. As recommended by the MIQE guidelines [48], the expression ratios were normalized with two internal reference genes encoding *actin* and *elongation factor 1α* (EF1α) from *M. persicae* and were calculated using the threshold cycle (2^-ΔΔCT^) method [49].

### 4.7. Analysis of Aphid Feeding Behavior

The EPG experiments were conducted on N4 nymphs. To that end, N1/N2 nymphs were fed for 48 h on Sty01-siRNA, Sty03-siRNA or NC-siRNA artificial diets. Two independent biological replicates were performed for each treatment. In total, 18 to 21 RNAi-treated aphids were placed individually on *Arabidopsis thaliana* plants and their feeding behavior was monitored for 8 h using EPG [10,30]. Each plant was used only once and replaced for each aphid. The aphids were attached to the gold wire of an insect electrode using water-based silver conductive paint glue (EPG Systems, Wageningen, Netherlands). Wired aphids were kept suspended on air for a fasting period, and then placed on the adaxial side of a leaf and connected to the EPG device. Their feeding behavior was recorded by a Giga-8 DC-EPG device using a Faraday cage to prevent electrical perturbation (EPG Systems, Wageningen, The Netherlands). The EPG data were acquired and analyzed using Stylet+ software for Windows (EPG Systems). The EPG-Excel Data Workbook developed by Sarria et al. [50] was used to process the following EPG variables: Waveform np (non-probing behavior), C (intercellular apoplastic stylet pathway when stylets penetrate leaf tissue), pd (potential drop corresponding to short intracellular puncture), E1 (continuous salivation into phloem sieve elements before phloem ingestion), and E2 (passive phloem sap uptake from the sieve elements). The variables were calculated as described by Backus et al. [51]; PPW (proportion of individuals that produced a specific waveform type), NWEI (number of waveforms per insect), WDI (waveform duration per insect), and WDEI (waveform duration per insect and per event).

### 4.8. Aphid Life Table Statistics

The impacts of the siRNA treatments on aphid fitness and on stylet-binding capacity were evaluated on adult aphids to ensure that stylets remained unchanged throughout the entire experiment, and to facilitate dissection of their mouthparts. To that end, N3 synchronized nymphs were fed for 48 h on siRNA artificial diets. When they became one-day old wingless adults, these adults were then placed individually in a clip cage on the adaxial side of six-week-old eggplant leaves. For each silencing treatment, the survival of 25 to 30 aphids was followed over a 15-day period. In addition, 10 individuals alive throughout the recording period were followed for nymph production. Newborn nymphs were counted daily and removed over the same period.

### 4.9. Statistical Analysis

All statistical analyses were carried out with R software (v. 3.4.1, [52]), with values of *p* < 0.05 considered as being statistically significant. Stylet labeling was compared using generalized linear models (GLM with binomial error, logit link function) and the chi-square test. Post-hoc pairwise comparisons were made using Tukey contrasts (*multcomp* package in R). The differences in transcript levels and EPG variables were analyzed using Welch’s *t*-test (Gaussian data) or a Mann–Whitney *U* test (non-Gaussian data). Statistical significance for the transcript levels and fecundity was assessed by one-way analysis of variance (ANOVA). The results of each statistical test are indicated in the figure legends or in Supplemental Tables S1 and S2. Note that all bars in Figure 2 and Figure 4 represent the standard deviation of the row data and should not be interpreted as the standard error of the estimated means.

## Data Availability

Data are contained in this article or Supplementary Materials.

## Supplementary Materials

The following supporting information can be downloaded at: https://www.mdpi.com/article/10.3390/ijms232315337/s1, Figure S1: Detection of effector–stylin interactions using the yeast two-hybrid system; Figure S2: Production of mature Mp10 proteins in *E. coli*; Table S1: Feeding behavior of *M. persicae* adults previously fed for 72 h on siRNA targeting *stylin-01* or *stylin-03* gene recorded on *Arabidopsis thaliana*. Non-sequential EPG variables; Table S2: Feeding behavior of *M. persicae* adults previously fed for 72 h on siRNA targeting *stylin-01* or *stylin-03* gene recorded on *Arabidopsis thaliana*. Sequential EPG variables; Table S3: List of oligonucleotides used in this study; Supplementary information: Material and Methods, modification of original plasmids to construct pLexAN-GG & pGADT7-GG.
