# Supplementary material for: Role of Acrostyle Cuticular Proteins in the Retention of an Aphid Salivary Effector
Source: Int J Mol Sci. 2022 Dec 5;23(23):15337. doi: 10.3390/ijms232315337 (PMC9736059; doi:10.3390/ijms232315337)
Supplement: Supplementary file 1 [file ijms-23-15337-s001.zip › ijms-2036297-supplementary.pdf]

Supplementary Materials:

Supporting Online Material for

Role of Acrostyle Cuticular Proteins in the Retention of an Aphid Salivary Effector

Maëlle Deshoux [1], Baptiste Monsion [1], Elodie Pichon [1], Jaime Jiménez [2], Aranzazu Moreno [2], Bastien Cayrol [1], Gaël Thébaud [1], Sam T. Mugford [3], Saskia A. Hogenhout [3], Stéphane Blanc [1], Alberto Fereres [2,*] and Marilyne Uzest [1,*]

Corresponding authors:
Email: afereres@ica.csic.es; marilyne.uzest@inrae.

Supplementary Figures

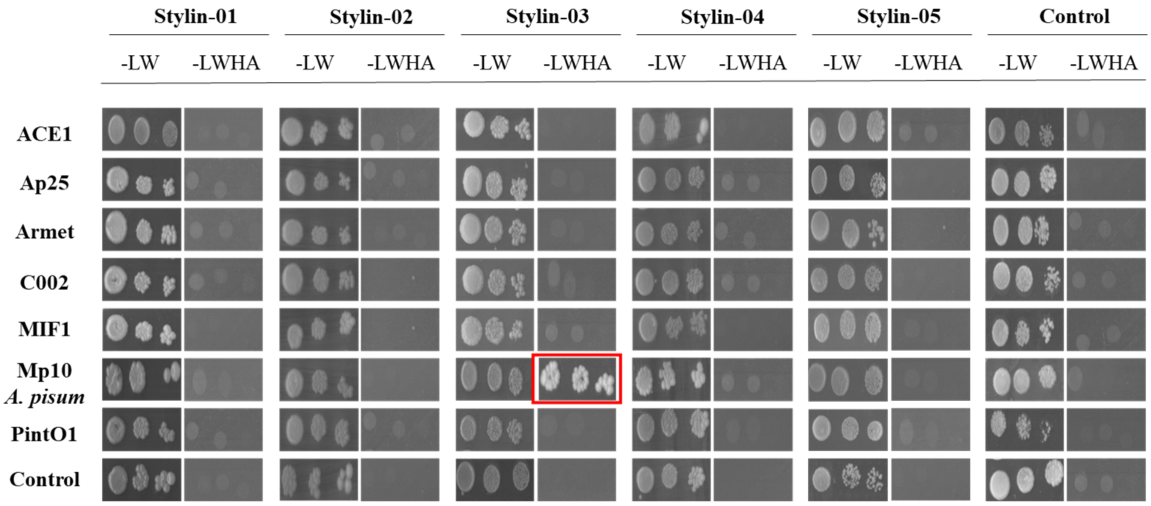

Figure S1. Detection of effector-stylin interactions using the yeast-two-hybrid system. Pairwise yeast-two hybrid assay of protein-protein interactions between effectors (activation domain fusions) and stylins (binding domain fusions) of *Acyrthosiphon pisum*. Controls are the two plasmids pGADT7-GG-6His and pLexAN-GG-6His. Yeasts cultures co-transformed with the two plasmids were adjusted to an OD600 of 0.2, diluted 1/10, 1/100, 1/1000 and spotted on a non-selective SD medium lacking leucine, and tryptophan [-LW] to control yeast growth, and on a selective SD medium lacking leucine, tryptophan, histidine and adenine [-LWHA] to detect yeasts in which interaction between effector and stylin occurs. On [-LWHA] medium, yeast growth was detected only for Mp10 vs Stylin-03 (red rectangle), but not for the other combinations. Yeast transformations and interaction analyses were performed twice with identical results. Photos were taken after 4 days of growth at 28°C.

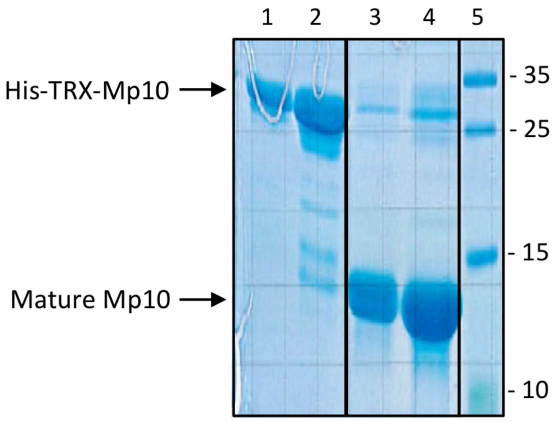

Figure S2. Production of mature Mp10 proteins in *E. coli*. Mp10_Apisum and Mp10_Mpersicae proteins were produced without their signal peptide in fusion with a histidin tag and the Thioredoxin protein. His-TRX-Mp10 fusions were purified by affinity chromatography on Ni-nitrilotriacetic resin Ni-NTA (Qiagen, Hilden, Germany): Samples were analyzed by SDS-PAGE 15% and stained with Coomassie Blue. His-TRX-Mp10_Apisum, lane 1; His-TRX-Mp10_Mpersicae, lane 2; Mp10_Apisum, lane 3 and Mp10_Mpersicae, lane 4. Mature proteins were obtained after cleavage and separation from His-TRX using AcTEV protease (Thermo Fisher Scientific, Rockford IL, USA) and elution from Ni-NTA column. Lane 5, prestained protein ladder (Thermo Fisher Scientific, Rockford IL, USA), with the protein sizes indicated in kDa (right). Expected size for His-TRX fused Mp10 and mature Mp10 without signal peptides are indicated.

Supplementary Tables

Table S1. Feeding behavior of *M. persicae* adults previously fed for 72 h on siRNA targeting *stylin-01* or *stylin-03* gene recorded on *Arabidopsis thaliana*. Non-sequential EPG variables (mean values ± standard error) for Sty01-siRNA-treated aphids (Sty01) and Sty03-siRNA-treated (Sty03) aphids compared to control aphids (NC).

	Aphids	PPW	NWEI	p	WDI	p	WDEI	p
Np								
	Sty01	19/19	17.47±1.97	0.339	4221.36±1016.85	0.525	227.68±45.58	1.000
	NC	19/19	20.58±2.53		4004.32±691.94		226.17±42.37	
	Sty03	21/21	12.48±1.84	0.247	2591.16±439.52	0.568	222.86±31.42	0.749
	NC	18/18	15.78±2.32		3484.47±734.50		250.69±55.51	
Probe								
	Sty01	19/19	17.32+1.92	0.325	24577.43±1016.82	0.525	1859.37±1559.34	0.686
	NC	19/19	20.47±2.52		24794.68±691.94		2015.94±1494.75	
	Sty03	21/21	12.43+1.84	0.247	26207.84±439.52	0.568	4407.21±6266.74	0.223
	NC	18/18	15.61±2.30		25314.53±734.50		3573.37±6204.97	
C								
	Sty01	19/19	21.26±2.13	0.374	13544.29±1136.60	0.566	695.51±63.22	0.931
	NC	19/19	24.37±2.71		14460.56±1097.78		703.12±86.62	
	Sty03	21/21	15.38±2.19	0.184	13447.41±1285.88	0.528	1002.96±70.20	0.183
	NC	18/18	18.94±2.38		14670.68±1432.42		1132.36±306.99	
pd								
	Sty01	19/19	155.53±12.39	0.270	732.15±58.11	0.337	4.70±0.07	0.871
	NC	19/19	178.37±16.22		821.62±71.20		4.68±0.09	
	Sty03	21/21	131.05±14.20	0.320	680.78±72.56	0.521	5.28±0.10	0.026
	NC	18/18	150.78±13.49		744.84±64.91		4.98±0.07	
II-1								
	Sty01	19/19	155.53±12.39	0.270	298.84±24.93	0.952	1.90±0.14	0.390
	NC	19/19	178.37±16.22		294.65±30.85		1.99±0.13	
	Sty03	21/21	131.05±14.20	0.320	272.16±29.50	0.385	2.19±0.06	0.530
	NC	18/18	150.78±13.49		307.59±27.42		2.13±0.04	
II-2								
	Sty01	19/19	155.53±12.39	0.270	168.16±12.26	0.318	1.14±0.03	0.040
	NC	19/19	178.37±16.22		148.63±15.06		1.01±0.07	
	Sty03	21/21	131.05±14.20	0.320	175.44±18.72	0.776	1.41±0.04	0.010
	NC	18/18	150.78±13.49		182.37±15.29		1.28±0.04	
II-3								
	Sty01	19/19	155.53±12.39	0.270	207.86±18.58	0.700	1.39±0.05	0.852
	NC	19/19	178.37±16.22		197.09±20.67		1.33±0.09	
	Sty03	21/21	131.05±14.20	0.320	188.92±20.98	0.623	1.51±0.05	0.103
	NC	18/18	150.78±13.49		202.65±18.15		1.41±0.03	
E1								
	Sty01	18/19	4.26±0.64	0.858	320.47±51.44	0.099	74.19±7.39	0.046
	NC	18/19	3.79±0.57		212.05±38.12		56.11±4.65	
	Sty03	19/21	3.38±0.63	0.682	205.91±41.47	0.921	66.58±6.81	0.093
	NC	17/18	3.72±0.66		197.83±40.73		51.44±6.41	
E2								
	Sty01	18/19	3.79±0.58	0.918	10447.41±1546.50	0.608	4769.65±1552.64	0.938
	NC	18/19	3.47±0.54		9306.55±1568.87		5210.88±1375.08	
	Sty03	19/21	3.14±0.57	0.754	12005.06±1772.49	0.418	8411.84±2061.88	0.317
	NC	16/18	3.44±0.63		9928.69±1795.18		5423.90±1779.13	
Number of single E1								
	Sty01	19/19	0.32±0.17	0.209				
	NC	19/19	0.47±0.14					
	Sty03	21/21	0.28±0.11	0.423				
	NC	18/18	0.24±0.14					

PPW, proportion of individuals that produced a specific waveform type; **NWEI**, number of waveform events per insect; **WDI**, waveform duration (s) per insect; **WDEI**, waveform duration (s) per event and per insect; Non-probe **Np**, non-probe activity; **Probe**, probe activity. Waveforms: **C**, intercellular stylet pathway; **pd**, short intracellular punctures; **II-1**, pd sub-phase II-1, **II-2**, pd sub-phase II-2, **II-3**, pd sub-phase II-3; **E** shows phloem-related activities: **E1**, correlates with salivation into phloem sieve elements; **E2**, regarded as ingestion from phloem.

P-values according to a Student *t*-test for Gaussian variables and Mann Whitney U test for non-Gaussian variables. Underlined numbers indicate significant differences ($p \leq 0.05$).

Table S2. Feeding behavior of *M. persicae* adults previously fed for 72 h on siRNA targeting *stylin-01* or *stylin-03* gene recorded on *Arabidopsis thaliana*. Sequential EPG variables (mean values ± standard error) for Sty01-siRNA-treated aphids (Sty01) and Sty03-siRNA-treated (Sty03) aphids compared to control aphids (NC).

Sequential variables	Aphids	PPW	WDEI	p
Time from start of EPG to first probe				
	Sty01	19/19	363.32±72.24	0.603
	NC	19/19	426.05±88.86	
	Sty03	21/21	301.51±43.75	0.234
	NC	18/18	439.64±69.84	
Time from the beginning of the first probe to first pd				
	Sty01	19/19	279.66±118.35	1.00
	NC	19/19	444.49±169.02	
	Sty03	21/21	212.48±81.72	0.443
	NC	18/18	285.99±127.20	
Time from the beginning of that probe to first E				
	Sty01	19/19	2094.57±252.15	0.542
	NC	19/19	2527.87±451.50	
	Sty03	21/21	3082.26±435.60	1.00
	NC	18/18	2899.43±344.33	
Duration of E1 followed by the first E2				
	Sty01	18/19	91.27±16.87	0.104
	NC	18/19	61.36±11.08	
	Sty03	19/21	79.02±15.34	<u>0.037</u>
	NC	16/18	56.65±12.56	
Duration of E1 followed by first sustained E2 (>10 min)				
	Sty01	16/19	87.56±17.21	0.707
	NC	16/19	50.79±3.76	
	Sty03	18/21	62.52±5.66	<u>0.015</u>
	NC	14/18	42.41±5.00	

PPW, proportion of individuals that produced a specific waveform type; **WDEI**, waveform duration (s) per event and per insect; **Probe**, probe activity. Waveforms: **pd**, short intracellular punctures: E shows phloem-related activities: **E1**, correlates with salivation into phloem sieve elements; **E2**, regarded as ingestion from phloem.

P-values according to a Student *t*-test for Gaussian variables and Mann Whitney *U* test for non-Gaussian variables. Underlined numbers indicate significant differences (p≤0.05).

Table S3. List of oligonucleotides used in this study.

Primers' name	Sequence (5′ to 3′)	Comments
**Primers for yeast two-hybrid constructs**		
Sty01-Mp-For	taagctcttctagcTACCCCGCTGGACAGAGC	Stylin-01 from *M. persicae*
Sty01-Mp-Rev	taagctcttcctcaTACCCCGCTGGACAGAGC	
Sty02-Mp-For	taagctcttctagcTACCCCGCCCAAGGTCCC	Stylin-02 from *M. persicae*
Sty02-Mp-Rev	taagctcttcctcaCTGGTACTGGGGTTCGGGT	
Sty03-Mp-For	taagctcttctagcCAACAGTATCAACCCACAACTCCTAT	Stylin-03 from *M. persicae*
Sty03-Mp-Rev	taagctcttcctcaCTACTTAATTTTGTTGTAAGCTGGTTCC	
Sty04-Mp-For	taagctcttctagcCAGACGGTGCAACCAGCACC	Stylin-04 from *M. persicae*
Sty04-Mp-Rev	taagctcttcctcaTTTGTTGAATTGTGGTGGTTCTAGTTTG	
Sty01-Ap-For	taagctcttctagcTACCCCGCATCACTGAACCC	Stylin-01 from *A. pisum*
Sty01-Ap-Rev	taagctcttcctcaCTGGTATTTTGGTTCGGGGG	
Sty02-Ap-For	taagctcttctagcTACCCCGCTCAAGGCCCC	Stylin-02 from *A. pisum*
Sty02-Ap-Rev	taagctcttcctcaCTGGTACTGTGGTTCGGGTGT	
Sty03-Ap-For	taagctcttctagcCAACAGTTTCAACCCACGACTC	Stylin-03 from *A. pisum*
Sty03-Ap-Rev	taagctcttcctcaCTTAATTTTGTTGTAAGCTGGTTCCG	
Sty04-Ap-For	taagctcttctagcCAGACGGTGCAGCCTGCA	Stylin-04 from *A. pisum*
Sty04-Ap-Rev	taagctcttcctcaTTTGTTGAATTGTGGTGGTTCTAGTTTG	
Sty05-Ap-For	taagctcttctagcCAATTCCAATGCCCCAAGAAGAAC	Stylin-05 from *A. pisum*
Sty05-Ap-Rev	taagctcttcctcaTTGTTTTTTATTCTTGTTCTGTTGCT	
ACE1-For	taagctcttctagcGCAGGAATAAAAAACTGCTC	ACE1 from *A. pisum*
ACE1-Rev	taagctcttcctcaAGGTTGACTTGTTTCACCC	
Ap25-For	ttaagctcttctagcACAAAAACTGAAAAATCTGACAAAG	Ap25 from *A. pisum*
Ap25-Rev	taagctcttcctcaTTTTTTTGACGATTTATTGTTATT	
Armet-For	ttaagctcttctagcCAATCGCGAACATTTACCG	Armet from *A. pisum*
Armet-Rev	taagctcttcctcaTAACTCTTCCTTGACGTAAGATGGT	
C002-For	ttaagctcttctagcGATTGGTCTGCCGCTG	Armet from *A. pisum*
C002-Rev	taagctcttcctcaAAAACGTCGAAGGAAACTTCCAA	
MIF1-For	ttaagctcttctagcATGCCTCATTTCCGTTTGGAAA	MIF1 from *A. pisum*
MIF1-Rev	taagctcttcctcaTAACTCTTCCTTGACGTAAGATGGT	
Ap-Mp10-For	ttaagctcttctagcGCGCCGCAAAAAGATG	Mp10 from *A. pisum*
Ap-Mp10-Rev	taagctcttcctcaGAAGAGAAGAAAAAAGGTGTTGTC	
Mp-Mp10-For	ttaagctcttctagcGCGCCGCAAAAAGATG	Mp10 from *M. persicae*
Mp-Mp10-Rev	taagctcttcctcaAAATTTGACAACACCTTTTTTCTT	
PintO1-For	ttaagctcttctagcAGTCTCTACCAGCCGC	PintO1 from *A. pisum*
PintO1-Rev	taagctcttcctcaCAATAATTCACGATCGATG	

**Primers for cloning in the plasmid pETtrx1b**		
Xho-Mp-Mp10-For<br>Nco1-Mp-Mp10-Rev	atctccatggccGCGCCGCAAAAAGATG<br>ggtgctcgagttaAAATTTGACAACACCTTTTTTCTTC	Mp10 from *M. persicae*
Xho-Ap-Mp10-For<br>Nco1-Ap-Mp10-Rev	atctccatggccGCGCCGCAAAAAGATG<br>ggtgctcgagttaAAATTTAACAACCCCTTTTTTCTTCTCT	Mp10 from *A. pisum*
**Primers for RT-qPCR**		
qSty01_Mp_For<br>qSty01_Mp_Rev	ACATTTGTCGTATCGTCATTG<br>GCTGGGTGCTGAATCTT	Stylin-01 from M. persicae [1]
qSty03_Mp_For<br>qSty03_Mp_Rev	GATGGATCTTATAAATGGGCGT<br>GATTGGCTGGGATCTTGAG	Stylin-03 from *M. persicae*
qActin_Mp_For<br>qActin_Mp_Rev	CGTTACCAACTGGGACGATATC<br>GGGTTCAATGGAGCTTCTGTAA	Actin from *M. persicae* [1]
qEF1α_Mp_For<br>qEF1α_Mp_Rev	AAAATGGACAAACCCGTGAA<br>GCTGTATGGTGGTTCAGTAGAA	EF1-α from *M. persicae* [1]
**siRNA for silencing**		
NC-siRNA	Reference SR-CL00-005 (Kaneka Eurogentec S.A., Seraing, Belgium)	siRNA duplex negative control
Sty01-siRNA	AUCCCAUUCGAGAUCCAAG(+dTdT)	Targeting Stylin-01 mRNA (*M. persicae)*
Sty03-siRNA	CACCAAUCAGCGUGAAAUA(+dTdT)	Targeting Stylin-03 mRNA (*M. persicae)*

1. Webster, C.G., Pichon, E., van Munster, M., Monsion, B., Deshoux, M., Gargani, D., Calevro, F., Jimenez, J., Moreno, A., Krenz, B., Thompson, J.R., Perry, K.L., Fereres, A., Blanc, S., and Uzest, M. 2018. Identification of plant virus receptor candidates in the stylets of their aphid vectors. J. Virol. 92, e00432-18.

Material and Methods

Modification of original plasmids to construct pLexAN-GG & pGADT7-GG

Briefly, the multi-cloning sites (MCS) of pLexAN (Dualsystems Biotech, Zurich, Switzerland) and pGADT7 (Clontech, Palo Alto, CA, USA) plasmids were removed and replaced by an insert containing ccdB and chloramphenicol resistance ORFs (as in Gateway plasmids), flanked by SapI recognition site producing cohesive ends (AGC and TGA) corresponding to serine and stop codons, respectively.

1) Deletion of SapI restriction site in pLexAN and pGADT7 plasmids

For both plasmids, a SapI restriction site close to the plasmid origin of replication (ColE1) was first removed by PCR mutagenesis using Phusion polymerase in GC buffer supplemented with 4% DMSO and specific primers:
ColE1>: 5′-CCTCGCTCACTGACTCGCTG-3′
MutSapI-ColE1<: 5′-AAGCGGAAGtGCGCCCAATAC-3′
followed by purification, DpnI treatment, phosphorylation and ligation steps.

2) pLexAN-GG plasmid

Sequences corresponding to ccdB and CmR ORFs (insert) were amplified from a former GoldenGate plasmid (Gateway-based vector) by PCR using Phusion polymerase and the two following primers:
SacI-SapI-K7ccdB>: 5′-AGTAGAGCTCAGCCGAAGAGCTTAGGCACCCCAGGCTT-3′
Stop-SapI-K7ccdB<: 5′-ATTATCACGAAGAGCTGTGTATAAGGGAGCCTGACA-3′
SapI-free pLexAN plasmid digested by SacI and SmaI was ligated with the PCR product digested by SacI, and transformed in *E. coli* DB3.1 strain. Colonies were screened on LB agar plates supplemented with kanamycin (50 μg/ml final concentration) and chloramphenicol (25 μg/ml final concentration).

3) pGADT7-GG plasmids

Sequences corresponding to ccdB and CmR ORFs (insert) were amplified from GoldenGate plasmid by PCR using Phusion polymerase and the two following primers:
Ser-SapI-K7ccdB> 5′-GCCGAAGAGCTTAGGCACCCCAGGCTT-3′
XhoI-SapI-K7ccdB<5′-CGATTCTCGAGTTATCACGAAGAGCTGTGTATAAGGGAGCCTGACA-3′
SapI-free pGADT7 plasmid was digested by NdeI, and cohesive extremities were filled-in using Klenow and dNTPs. This plasmid was then restricted with XhoI and ligated with the insert digested by XhoI, and transformed in *E. coli* DB3.1 strain. Colonies were screened as described above.

4) Cloning stylin genes and effector sequences in pLexAN-GG and pGADT7-GG

To clone *stylin* genes or effector sequences flanked with SapI restriction sites into modified pLexAN-GG and pGADT7-GG, a GoldenGate reaction was performed using SapI and T4 DNA ligase. GoldenGate (GG) cloning is seamless. Recombinant plasmids resemble original ones and just lack the MCS. We took advantage of the positive selection of recombinant clones using ccdB in modified vectors.
